# Replacing a Native *Wolbachia* with a Novel Strain Results in an Increase in Endosymbiont Load and Resistance to Dengue Virus in a Mosquito Vector

**DOI:** 10.1371/journal.pntd.0002250

**Published:** 2013-06-06

**Authors:** Guowu Bian, Guoli Zhou, Peng Lu, Zhiyong Xi

**Affiliations:** 1 Department of Microbiology and Molecular Genetics, Michigan State University, East Lansing, Michigan, United States of America; 2 Department of Parasitology, Zhongshan School of Medicine, Key Laboratory of Tropical Disease Control, Ministry of Education, Sun Yat-Sen University, Guangzhou, Guangdong, China; 3 Sun Yat-sen University - Michigan State University Joint Center of Vector Control for Tropical Diseases, Guangzhou, Guangdong, China; Monash University, Australia

## Abstract

*Wolbachia* is a maternally transmitted endosymbiotic bacterium that is estimated to infect up to 65% of insect species. The ability of *Wolbachia* to both induce pathogen interference and spread into mosquito vector populations makes it possible to develop *Wolbachia* as a biological control agent for vector-borne disease control. Although *Wolbachia* induces resistance to dengue virus (DENV), filarial worms, and *Plasmodium* in mosquitoes, species like *Aedes polynesiensis* and *Aedes albopictus*, which carry native *Wolbachia* infections, are able to transmit dengue and filariasis. In a previous study, the native *w*PolA in *Ae. polynesiensis* was replaced with *w*AlbB from *Ae. albopictus*, and resulted in the generation of the transinfected “MTB” strain with low susceptibility for filarial worms. In this study, we compare the dynamics of DENV serotype 2 (DENV-2) within the wild type “APM” strain and the MTB strain of *Ae. polynesiensis* by measuring viral infection in the mosquito whole body, midgut, head, and saliva at different time points post infection. The results show that *w*AlbB can induce a strong resistance to DENV-2 in the MTB mosquito. Evidence also supports that this resistance is related to a dramatic increase in *Wolbachia* density in the MTB's somatic tissues, including the midgut and salivary gland. Our results suggests that replacement of a native *Wolbachia* with a novel infection could serve as a strategy for developing a *Wolbachia*-based approach to target naturally infected insects for vector-borne disease control.

## Author Summary

*Aedes polynesiensis* is a vector for both dengue and filariasis in the South Pacific. Efforts are ongoing to utilize *Wolbachia* as a biological control agent targeting this vector through either population suppression via releases of incompatible males or population replacement for spreading disease resistance into a population. Replacing the native *Wolbachia* with a novel infection from *Ae. albopictus* has generated the “MTB” strain of *Ae. polynesiensis*. This MTB mosquito is reproductively-incompatible with the wild type of *Ae. polynesiensis* and has a low susceptibility for filarial worms. In this work, we show that the MTB mosquito is resistant to dengue virus with a reduced viral infection in the mosquito whole body, midgut, head, and saliva. Our results further show its refractoriness to dengue virus is associated with a dramatic increase in *Wolbachia* density in those mosquito tissues where dengue virus needs to reside, replicate, and travel in order to be transmitted to humans. These results suggest that the MTB strain has the potential to be used in *Wolbachia*-based strategies to control both dengue and filariasis in the South Pacific.

## Introduction

*Wolbachia* is a maternally transmitted endosymbiotic bacterium that infects an estimated 65% of insect species [1], [2]. The ability of *Wolbachia* to both induce viral interference and spread into mosquito vector populations makes it a potential candidate as a biological control agent for dengue control [3], [4], [5], [6], [7]. The primary dengue vector, *Aedes aegypti*, does not naturally carry *Wolbachia*. When artificially introduced into *Ae. aegypti*, three types of *Wolbachia* (*w*AlbB, *w*MelPop-CLA, and *w*Mel) show significant inhibition of dengue virus (DENV) replication and dissemination, resulting in either complete or partial block of viral transmission [4], [6], [7]. Furthermore, this *Wolbachia*-mediated pathogen interference has a broad spectrum and can inhibit a variety of pathogens in *Ae. aegypti*, including Chikungunya, *Plasmodium*, and filarial worms [7], [8].

In addition to *Ae. aegypti*, dengue is transmitted by secondary mosquito vectors including *Ae. albopictus* and *Ae. polynesiensis* [9], [10], [11]. Both species of mosquitoes can transmit other pathogens to humans as well. While *Ae. albopictus* is a competent vector for at least 22 arboviruses [10], *Ae. polynesiensis* is the primary vector of *Wuchereria bancrofti* in the South Pacific [12]. In contrast to *Ae. aegypti*, both of them naturally carry *Wolbachia* infections. *Ae. albopictus* is infected with *w*AlbA and *w*AlbB [13], and *Ae. polynesiensis* with *w*PolA [14]. These *Wolbachia* can induce cytoplasmic incompatibility (CI) when infected males mate with females that are uninfected or carry different type of *Wolbachia* [14], [15]. Although native *Wolbachia* infections were reported to confer host resistance to pathogen infection in both the *Drosophila* and *Culex* mosquitoes [16], [17], neither *w*AlbA and *w*AlbB, nor *w*PolA appear to induce resistance to DENV in *Ae. albopictus* or *Ae. polynesiensis*, respectively [6], [9], [18].

*Wolbachia*-induced viral interference depends on the density of *Wolbachia*. In the original *Ae. albopictus* Aa23 cell line, *w*AlbB reaches a density high enough to completely clear dengue infection [18]. A strong negative linear correlation was observed between the genome copy numbers of *w*AlbB and DENV [18]. In the mosquito vector, this density-dependent viral inhibition occurs in a tissue-specific manner. A very low density of native *Wolbachia* in mosquito somatic tissues (e.g., midguts and salivary glands) makes its resistance to DENV undetectable [18], or detected with a weak effect [19]. When the native *Wolbachia* was replaced with a *w*Mel infection, the transinfected *Ae. albopictus* becomes strongly resistant to DENV. This resistance is associated with a 7-fold increase in *w*Mel density compared to the native infection [20].

Following ingestion by mosquitoes in a DENV-infected blood meal, virus first enters the midgut epithelial cells and infects the midgut tissue, in which it replicates to produce more viral particles. DENV then escapes from the midgut and disseminates through the hemolymph to other tissues, including the salivary glands, where it replicates and resides until it is injected into a human host [21]. During this journey virus needs to pass a number of physiological barriers, such as the midgut infection/escape barrier and salivary gland infection/escape barrier, and is subjected to a series of attacks by the mosquito immune system [21], [22], [23], [24]. The dynamics and tropism of DENV within a mosquito are dependent on the mosquito strain, the virus genotype, and environmental factors [25]. Recent studies show that endogenous microbial flora, including *Wolbachia*, also influence the mosquito's tissue- and cell-specific susceptibility to DENV by boosting basal immunity [22], [26].

Due to its importance in transmitting both Lymphatic filariasis and dengue in the South Pacific, *Ae. polynesiensis* was targeted for control through either population suppression via releases of incompatible males or population replacement to harness CI as a gene-drive mechanism for spreading disease resistance into a population [12], [14]. A strain of *Ae. polynesiensis* was previously infected with *Wolbachia* from *Ae. riversi* through interspecific hybridization and introgression. This strain was used to test a population suppression strategy in semi-field conditions with encouraging results [12]. With successful experiences in *Wolbachia* transinfection through embryo microinjection, additional studies were conducted to transfer *Wolbachia* from *Ae. albopictus* into the aposymbiotic strain of *Ae. polynesiensis* (APMT), resulting in the “MTB” strain stably infected by *w*AlbB only [14]. There is a strong bi-directional CI when the MTB strain crosses with the wild type “APM” strain. Furthermore, the number of successfully developing infective stage filarial worms was reduced in the MTB strain [14], which was consistent with previous findings that interaction of *w*AlbB with mosquito hosts can trigger a process of pathogen interference [26].

The mosquito MTB strain has a decreased ability to regulate oxidative stress [14]. We previously showed that *Wolbachia* can induce reactive oxygen species (ROS)-dependent activation of the Toll pathway to control DENV in *Ae. aegypti* [26]. Due to the role of *Ae. polynesiensis* in dengue transmission, it is important to know how *w*AlbB will influence MTB's susceptibility for DENV. In this study, we measured the vector competence of the MTB strain for DENV serotype 2 (DENV-2) as compared to the naturally infected APM strain of *Ae. polynesiensis*. We also compared the *Wolbachia* density between the MTB and APM strain. These results indicate that *w*AlbB induces a strong resistance to DENV in the MTB strain, which is associated with an increased *Wolbachia* density in mosquito somatic tissues.

## Methods

### Ethics statement

This study was performed in strict accordance with the recommendations in the Guide for Care and Use of Laboratory Animals of the National Institutes of Health. The protocol was approved by the Institutional Animal Care and Use of the Michigan State University (Application #: 03/11-040-00).

### Mosquito rearing and cell culture maintenance

All mosquito strains used in these experiments, including the wild-type APM strain and the transfected line MTB of *Ae. polynesiensis*, were maintained on sugar solution at 27°C and 85% humidity with a 12-hr light/dark cycle according to standard rearing procedures. After transinfection of *w*AlbB into AMPT, MTB females were out-crossed with AMPT males for six consecutive generations [14]. APM and MTB were reared in a regular condition for at least two generations before used for experiments with the goal to recover and re-colonize gut bacteria [7]. Female mosquitoes, 3–5 d after eclosion, were fed on the blood of anesthetized white mice to initiate egg development. The *Ae. albopictus* cell line C6/36 was grown in minimal essential medium (MEM) with 10% heat-inactivated FBS, 1% L-glutamine, and 1% non-essential amino acids at 32°C and 5% CO_2_.

### DENV-2 infections

The New Guinea C strain of DENV-2 was propagated in C6/36 cells according to standard conditions [27]: In brief, 0.5-ml aliquots of virus stock were used to infect 75-cm^2^ flasks of C6/36 cells, at 80% confluence, with a multiplicity of infection (MOI) of 3.5 virus particles/cell. Infected cells were incubated for 7 days. Then, cells were harvested with a cell scraper and lysed by repeated freezing and thawing in dry ice and a 37°C water bath. Virus isolated from these cells was combined with the supernatant [28], resulting in virus suspension with a titer of 2×10^7^ PFU/ml. For infection through intrathoracic injection, 69 nl of the above virus suspension was used for injection into each female. For infection through oral feeding, the resulting virus suspension was mixed 1∶1 with commercial human blood. A flask with uninfected C6/36 cells was maintained under similar conditions and used to create the noninfectious blood meal that served as our control. The blood meal was maintained at 37°C for 30 min prior to use for feeding 7-day-old mosquitoes [28].

### Mosquito dissections

Mosquitoes were dissected to collect the midguts in RNALater at 4, 7 and 10 days post infection (dpi) and thorax at 14 dpi, with three individual mosquitoes in a single replicate. At least five replicate biological assays were performed. Total RNA was extracted using the RNeasy kit (QIAGEN). To measure the virus titers in mosquito bodies, at 14 days after a blood meal, mosquitoes were briefly washed in 70% ethanol, then rinsed in sterile distilled water. The midgut and thorax were dissected in sterile PBS and transferred separately to microcentrifuge tubes containing 150 µl of MEM, and then homogenized with a Kontes pellet pestle motor in a sterile environment. To measure the density of *Wolbachia* in mosquito tissues, the midguts, salivary glands, fat bodies, and ovaries of 7-day-old non-blood-fed females were dissected and transferred separately to microcentrifuge tubes containing 50 µl of STE buffer for extraction of total genomic DNA.

### Real-time qPCR assays

To measure the number of viral genome copies, total virus RNA was extracted using the RNeasy kit (QIAGEN) and reverse-transcribed using Superscript III (Invitrogen, Carlsbad, California, USA) with random hexamers. qRT-PCR was conducted using primers targeting the dengue NS5 gene and the host RPS6 [29]. The dengue genome copy number was normalized using the RPS6 results. Two recombinant plasmids containing the targeted fragments were diluted from 10^1^ to 10^8^ copies/reaction and used to generate separate standard curves for NS5 and RPS6. Real-time quantitation was performed using the QuantiTect SYBR Green PCR Kit (Qiagen) and ABI Detection System ABI Prism 7000 (Applied Biosystems, Foster City, California, USA). Three independent biological replicates were assayed, and all PCR reactions were performed in triplicate. To determine the number of copies of the *Wolbachia* genome, real-time PCR was carried out as previously described [22], [30].

### Plaque assays for DENV-2 virus titration

Virus titers in the tissue homogenates were measured as previously reported [28]: The virus-containing homogenates were serially diluted and inoculated into C6/36 cells in 24-well plates. After incubation for 5 days at 32°C and 5% CO_2_, the plates were assayed for plaque formation by peroxidase immunostaining, using mouse hyperimmune ascitic fluid (MHIAF, specific for DENV-2) and a goat anti-mouse HRP conjugate as the primary and secondary antibodies, respectively.

### Indirect immunofluorescence assay (IFA)

At 7 dpi, the viral antigen in the midguts of mosquitoes was detected by using an indirect IFA. Mosquito midguts were dissected in PBS and fixed in 4% paraformaldehyde for 5 h, and then incubated for 1 h at room temperature in a PBS-BSA-Triton solution (1× PBS, 1% Bovine Serum Albumin, and 0.1% Triton X-100), a mouse anti-dengue complex monoclonal antibody (obtained from the Centers for Disease Control, Atlanta, GA), and a fluorescein-conjugated affinity-purified secondary antibody (Millipore) were used in all midguts assays. Specimens were examined with a Zeiss (Germany) fluorescence microscope.

### Transmission assay

Mosquitoes that had been infected with DENV-2 as described above were maintained for 14 days for forced salivation assays. The assays were conducted as previously reported [31], [32]: In brief, mosquitoes were deprived of food for 24 h prior to forced salivation. The legs and wings of each mosquito were cut away, and the proboscis was inserted into 25 µl of feeding solution (50% FBS/164 mM NaCl/100 mM NaHCO_3_/0.2 mM ATP/≈50 µg sucrose/phenol red, pH 7.0) [32] in a 0.2-ml PCR tube. After 90 min, the mosquitoes were removed, and the feeding solution from each mosquito was sterilized by Millex-GV filter for plaque assays.

## Results

### A reduced viral infection in the whole bodies of MTB mosquitoes

**Figure 1:**
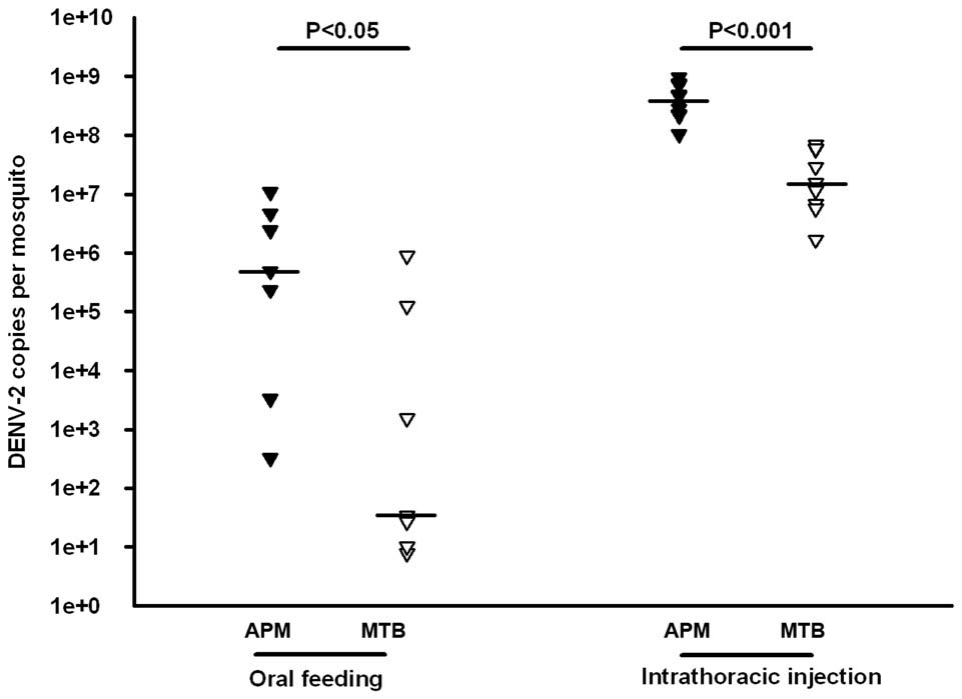
Inhibition of dengue infection in the whole bodies of MTB mosquitoes. At 14 dpi (days post infection) through a blood meal or intrathoracic injection, the whole bodies of MTB and APM mosquitoes were collected, and the number of genome copies of the DENV genome was determined by qRT-PCR using primers for the NS5 gene; the results were normalized to the *Ae. polynesiensis* ribosomal protein S6 (RPS6). Lines indicate the median of the ten biological replicates. Significance was determined using a Mann-Whitney U test.

The potential use of the MTB strain to control *Ae. polynesiensis* in a dengue endemic area provides a rationale to test if the MTB strain is resistant to DENV as compared to the wild type of *Ae. polynesiensis*. To test this, we first compared DENV-2 genome copies in the whole body between the MTB strain and the wild type APM strain at 14 days post infection (dpi). Mosquitoes were infected through either oral feeding with DENV-2 infected blood or intrathoracic injection with cell medium containing DENV-2. As a result, we observed that the median number of viral genomes in the whole bodies of MTB mosquitoes was 1.4×10^4^ times lower than that of APM mosquitoes when mosquitoes were infected through oral feeding (Mann-Whitney U test, P<0.05) (Fig. 1). Similarly, mosquito infection through intrathoracic injection led to a significantly lower viral infection in whole bodies of MTB mosquitoes as compared to APM mosquitoes (Mann-Whitney U test, P<0.001). There was a 28.3-fold reduction in the median number of viral genome copies in MTB mosquitoes comparing to APM mosquitoes (Fig. 1).

### An inhibited viral replication in the midguts of MTB mosquitoes

**Figure 2:**
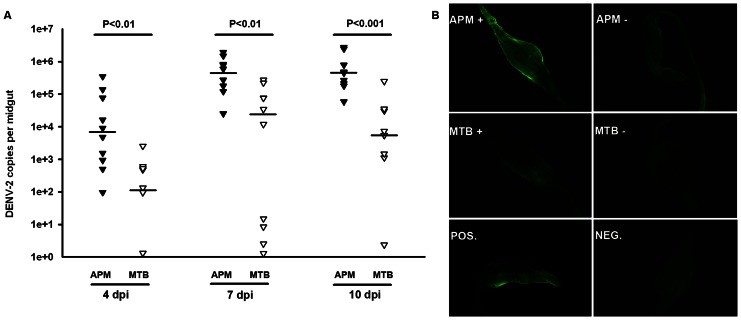
Inhibition of dengue infection in the midgut of MTB mosquitoes. (**A**) At 4, 7 and 10 dpi with a blood meal containing DENV-2, mosquito midguts were collected, and the number of genome copies of the DENV-2 genome was determined by qRT-PCR using primers for the NS5 gene; the results were normalized to the *Ae. polynesiensis* RPS6. Lines indicate the median of the ten biological replicates. Significance was determined using a Mann-Whitney U test. (**B**) At 7 dpi through a blood meal, mosquito midguts were collected and fixed, and the viral antigen was detected using a mouse anti-Dengue complex monoclonal antibody by indirect fluorescence assay (IFA). One representative midgut from each category is shown. APM mosquitoes fed with dengue-infected or -uninfected blood meal was used as positive (POS.) and negative (NEG.) control, respectively. +, dengue-positive; −, dengue-negative.

In order to determine how viral inhibition occurs in a tissue-specific manner in MTB mosquitoes, we examined whether or not viral replication in the midguts of MTB mosquitoes was suppressed. Both MTB and APM mosquitoes were fed with the DENV-2 infected blood and viral genome copy numbers in the midguts were measured at three different time points: 4, 7 and 10 dpi. The DENV-2 copy numbers were significantly lower at all of the three time points in the midguts of MTB mosquitoes than in APM mosquitoes (Mann-Whitney U test, P<0.01) (Fig. 2A). The viral infection in mosquito midguts at 7 dpi was further assayed by IFA to visualize the intensity and distribution of DENV-2 using an antibody against the viral envelop protein. Consistently, 76.2% (16/21) of APM midguts showed strong positive signals while only 10% (2/20) of MTB midguts had weak positive signals (Fisher's Exact Test, P<0.001) (Fig. 2B; Table 1). This indicates that viral replication is strongly inhibited in the midguts of MTB mosquitoes.

**Table 1:** *w*AlbB confers resistance to DENV-2 in both midguts and heads of MTB mosquitoes.

	% of infected samples		P value
Tissues	APM	MTB	
Midguts*	76.2 (16/21)	10.0 (2/20)	<0.001
Heads#	100 (30/30)	26.7 (8/30)	<0.001

* At 7 dpi through an infectious blood meal, the midguts of MTB and APM mosquitoes were collected and fixed, and the viral antigen was detected using a mouse anti-Dengue complex monoclonal antibody by indirect fluorescence assay (IFA).

# At 14 dpi, the heads of both mosquitoes were collected to extract the total RNA. DENV-2 was diagnosed by RT-PCR using primers for the NS5 gene. Amplification of *Ae. polynesiensis* RPS6 was used to verify the quality of the RNA samples. Significance was shown in Fisher's exact test.

### An inhibited viral dissemination to the heads of MTB mosquitoes

Following replication in the midgut, DENV-2 migrates to the mosquito head where it reaches a peak infection 14 dpi [25]. To test whether viral dissemination was affected in MTB mosquitoes, we assayed the viral infection at this time point by RT-PCR and compared the head infection rate between MTB and APM mosquitoes. As shown in Table 1, 100% (30/30) of APM heads were positive whereas viruses were only detected in 26.7% (8/30) of MTB heads (Fisher's Exact Test, P<0.001). This indicates the possibility that viral dissemination to mosquito heads is significantly reduced in MTB mosquitoes as compared with APM mosquitoes.

### Inhibition of viral transmission potential of MTB mosquitoes

**Figure 3:**
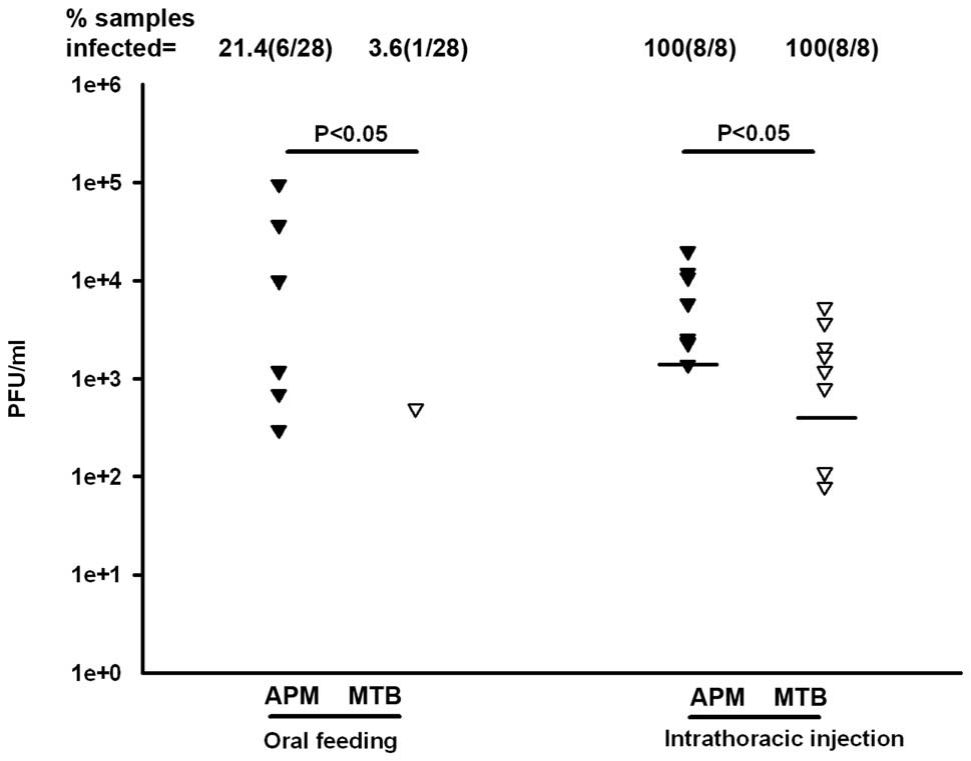
*In vitro* assay of DENV-2 transmission by MTB and APM mosquitoes. At 14 days after a blood meal containing DENV-2 or infection through intrathoracic injection, the wings and legs were removed, and the proboscis of each mosquito was inserted into a feeding solution for 90 min. Solution from each mosquito was analyzed for infectious DENV-2 by plaque assays. Lines indicate the log of the median values. Twenty eight and eight biological replicates were used in oral feedings and intrathoracic injection assays, respectively.

To determine whether viral transmission potential is reduced in MTB mosquitoes, we measured infectious viral particles in the saliva released during feeding from the proboscises of mosquitoes at 14 dpi by plaque assay. Mosquitoes were infected through either oral feeding with DENV-2 infected blood or by intrathoracic injection with cell medium containing DENV-2. When mosquitoes were infected through a bloodmeal, 21.4% (6/28) of the APM saliva samples were positive for the envelope protein of DENV-2, whereas DENV-2 was detected in 3.6% (1/28) of the MTB saliva samples (Chi-Square test, P<0.05) (Fig. 3). In the intrathoracic injection experiment, 100% (8/8) of saliva samples were positive for both mosquito strains, although the viral infection level was significantly lower in MTB than APM mosquitoes (Mann-Whitney U test, P<0.05) (Fig. 3). These results indicate MTB mosquitoes have a lower viral transmission potential than APM mosquitoes.

### A large increase in *Wolbachia* density in the somatic tissues of MTB mosquitoes

**Figure 4:**
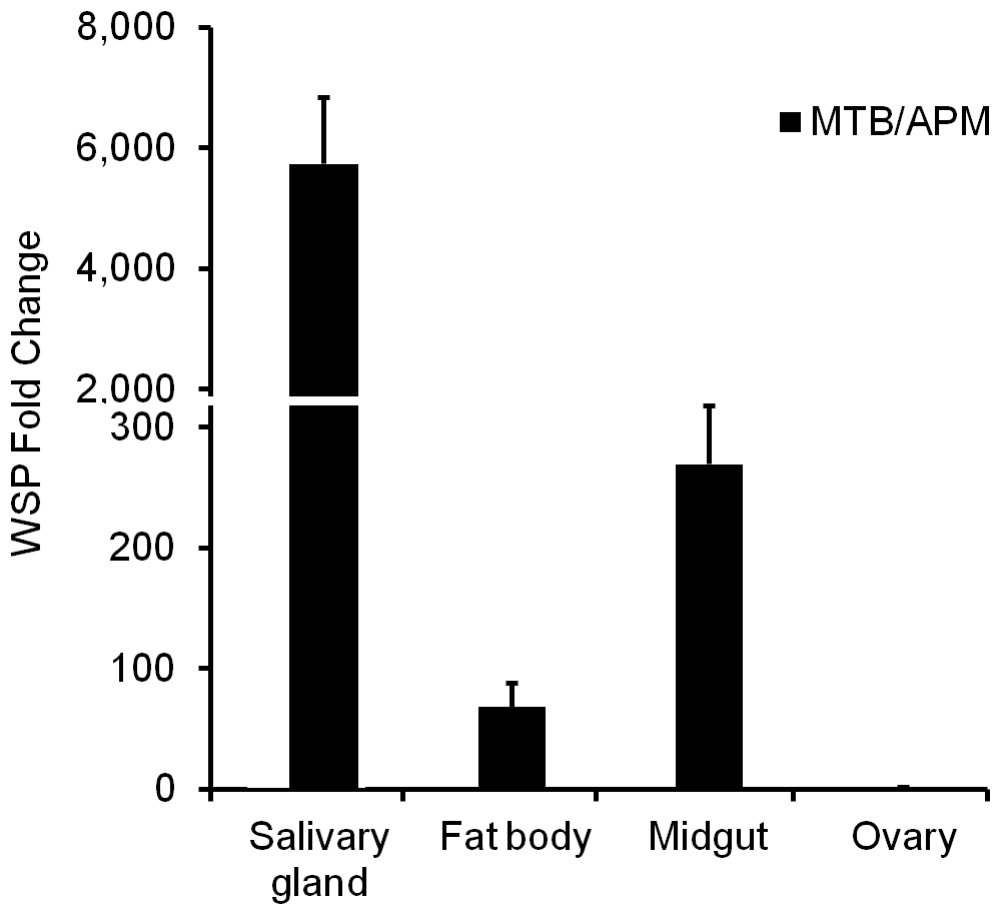
Resistance of MTB mosquitoes to DENV-2 is associated with a high density of *Wolbachia* in mosquito somatic tissues. The fold change in genome copy of the *Wolbachia* surface protein (WSP) gene in MTB mosquitoes is compared to APM mosquitoes. The copy number of the *Wolbachia* wsp was normalized by *Ae. polynesiensis* RPS6. In all the assays, the midguts, salivary glands, fat bodies, and ovaries of 7-day-old non-blood-fed females were dissected and used for extraction of total genomic DNA. Error bars are standard errors of the mean of twelve biological replicates.

We previously found that *Wolbachia* can induce resistance to dengue infection in a *Wolbachia*-density dependent and a tissue-specific manner [18]. To test whether the above viral inhibition is caused by an increased *Wolbachia* density in the somatic tissues of MTB mosquitoes, we compared the *Wolbachia* density between MTB and APM mosquitoes using qPCR. We found that the *Wolbachia* density is significantly higher in the salivary glands (5,738-fold), fat bodies (68-fold), and midguts (269-fold) of MTB mosquitoes as compared to APM mosquitoes, while no differences were observed in the ovaries (1-fold) of APM and MTB mosquitoes (Fig. 4). These results suggest that the high density of *Wolbachia* in somatic tissues of MTB mosquitoes may contribute directly to their low susceptibility for DENV.

## Discussion

The *Wolbachia* strain *w*AlbB is able to induce resistance to DENV in both mosquito vectors and cell lines [6], [18]. After the native *w*PolA was replaced with *w*AlbB in *Ae. polynesiensis*, we observed that *w*AlbB inhibits dengue viral replication in mosquito midguts and dissemination to mosquito heads. This artificial infection also reduces viral transmission potential through mosquito bites and suppresses the viral infection level in the whole bodies of mosquitoes. Our results further show that the density of *w*AlbB is 269-fold and 5,738-fold higher in the midguts and salivary glands, respectively, of MTB mosquitoes than the native *w*PolA infection in APM mosquitoes. This supports the previous finding that the strength of *Wolbachia*-mediated viral inhibition depends on the *Wolbachia* density [18].

Although it naturally carries a *Wolbachia* infection, *Ae. polynesiensis* is a compatible vector for both DENV and *Wuchereria bancrofti* in South Pacific [9], [12]. This is similar to *Ae. albopictus* and *Culex quinquefasciatus* in that the presence of the native *Wolbachia* infection in mosquitoes does not inhibit their abilities as vectors to transmit human pathogens. Evidence supports that *Wolbachia*-mediated viral inhibition occurs in a tissue-specific manner with the effect dependent on the *Wolbachia* density in the local tissue [18]. In a host cell with a very low level of *Wolbachia* infection, coexistence of *Wolbachia* and DENV can be observed in the cytoplasm [18]. Before a mosquito can transmit DENV to humans, viruses ingested by a mosquito in a blood-meal must sequentially migrate through and replicate in the midgut and salivary glands. We previously found that the *Wolbachia* density in both the midgut and salivary gland of *Ae. albopictus* was too low to induce a resistance to DENV [6], [18]. A similar situation may be true for *Ae. polynesiensis*. Consistent with this predication, the *Wolbachia* density in the midgut and salivary gland of the wild type APM strain is hundreds of times lower than that of the resistant MTB strain.

It is interesting to note that the above difference in the *Wolbachia* density between APM and MTB mosquitoes occurs only in the somatic tissues, and not the reproductive tissues (ovaries). This distribution pattern could be due to the strain of *Wolbachia* or the history of *Wolbachia*-host association. Previous studies showed a *Wolbachia* strain-specific distribution in insect hosts [33]. The native *Wolbachia* in APM mosquitoes may not be able to develop an infection at as high a level as *w*AlbB in somatic tissues. Alternatively, the native infection may have initially been at high levels, similar to *w*AlbB in the somatic tissues of MTB mosquitoes, but was gradually reduced to the current level in APM mosquitoes as *Wolbachia* and host co-adapted during evolution. Previous studies show a long-term attenuation of *w*MelPop in a non-native host, *Drosophila simulans* [34]. If *w*AlbB behaves similarly, it may compromise the effectiveness of this bacterium in pest control.

Our results indicate that *Wolbachia* in midguts can have a significant impact in the development of mosquito resistance to DENV. DENV has to pass the midgut infection barrier with a peroral infection. When infected by an intrathoracic injection, viruses bypass the midgut infection barrier and directly disseminate through the hemolymph. We observed a 1.4×10^4^ –fold reduction in the viral infection in the whole bodies of MTB mosquitoes as compared to those of APM mosquitoes with a peroral infection by DENV-2, while just a 28.3-fold reduction was observed for the same comparison with the viral intrathoracic injection. In addition, only 3.6% of MTB saliva was dengue-positive with the viral peroral infection, as compared to 100% of MTB saliva positive with the viral intrathoracic injection. The above results suggest that *Wolbachia*-induced viral interference in midguts may contribute to the majority of the virus-blocking effect in MTB mosquitoes.

The fact that *Wolbachia*-mediated resistance has a broad spectrum against a variety of pathogens in *Ae. aegypti* indicates that general killing mechanisms should be triggered by *Wolbachia* [7], [8], [16], [26]. Similarly, *w*AlbB is also found to suppress filarial worm loads in MTB mosquitoes as conducted by a parallel study in a recent publication [14]. Thus, *w*AlbB is the second example showing that *Wolbachia* can interact with mosquito hosts to inhibit both DENV and worm infection. We previously found that *Wolbachia* can induce ROS production, which activates the Toll pathway to control DENV in *Ae. aegypti* [26]. When the levels of H_2_O_2_ were compared before blood meal, there was a significantly higher level of ROS in MTB mosquitoes than in APM mosquitoes [14]. This suggests that a high density of *w*AlbB induces more oxidative stress in MTB mosquitoes than the native *w*PolA does in APM mosquitoes, consistent with our prediction that a high ROS production can contribute to the antiviral resistance [26].

Until now, *w*AlbB has been observed inducing resistance to DENV in the *Ae. albopictus* Aa23 cell line [18], *Ae. aegypti* [6], and *Ae. polynesiensis* mosquitoes. Among them, *w*AlbB is a native infection in Aa23 cells, but an artificial infection in *Ae. aegypti* and *Ae. polynesiensis*. It is important to note here that the native *w*AlbB infection can completely clear DENV in Aa23 cells [18]. This suggests that *w*AlbB has the ability to block DENV in a host cell when the cellular physiological environment is optimized for *w*AlbB to grow, such that it reaches a high infection level. Until now, both *w*Mel and *w*MelPop were reported to induce complete blockage of DENV transmission in *Ae. aegypti* [4], [7], while *w*AlbB induces a strong resistance to DENV with some viruses leaking from its inhibition in *Ae. aegypti* [6]. Considering that experimental methods, viral strains and mosquito strains are different in these studies, a direct comparison of viral interference and fitness between *w*AlbB and *w*Mel/*w*MelPop in the same host genetic background is needed to ascertain which *Wolbachia* strain is better suited for use in dengue control. From the disease control standpoint, an ideal strain of *Wolbachia* should be able to block pathogens in mosquitoes effectively enough to interrupt the disease transmission, and also be benign enough to the mosquito hosts that it can be persistently maintained in populations. It would be interesting to know if a perfect viral blockage is essential for disease control, and what extent of the host fitness cost associated with *Wolbachia* is acceptable.

Previous studies show *Wolbachia*-host interactions are determined by the *Wolbachia* strain, the host genotype, and the environment [35], [36], [37], [38]. It is still unclear why some native *Wolbachia* are unable to induce pathogen interference in their original hosts, although it appears related to their density. Although the artificial infections all reported inducing a resistance to pathogens [4], [6], [7], [8], this does not mean that any strain of *Wolbachia*, when introducing into a new mosquito host, will induce pathogen interference. We also cannot exclude the possibility that certain *Wolbachia*-host associations may even facilitate pathogen infection in an insect vector [39]. Those effects are determined by how a mosquito physiological system is perturbed or modified by its interaction with each specific strain of *Wolbachia*. The outcome becomes even more of a challenge to predict when considering that *Wolbachia*-host interactions may evolve over time. Despite these gaps in our knowledge, our results show replacement of native *Wolbachia* with a novel infection is a potentially practical way to develop pathogen resistant mosquitoes for modification of those vector populations.

Both the ability of *w*AlbB to induce resistance to filarial worms and DENV and the bi-directional nature of CI between *w*AlbB and *w*PolA in *Ae. polynesiensis* make it possible to develop MTB line as a tool to block disease transmission through population replacement. This result can be achieved by large-scale releases of MTB females. Alternatively, a strategy can be designed to start with population suppression by inundative releases of MTB males, followed by release of MTB females to initiate population replacement. Technically, the latter will be more acceptable to the public because males do not feed on human blood or transmit disease. When the population is then suppressed to a low density, the number of females that need to be released will be reduced. This would make population replacement happen parallel to a decrease in annoyance by mosquito biting. However, its deployment would require the development of a system with a high efficacy for mass rearing and sex separation, which is an ongoing effort in the field.
